# Deciphering microbial spatial organization: insights from synthetic and engineered communities

**DOI:** 10.1093/ismeco/ycaf107

**Published:** 2025-06-27

**Authors:** Estelle Pignon, Yolanda Schaerli

**Affiliations:** Department of Fundamental Microbiology, University of Lausanne, 1015 Lausanne, Switzerland; Department of Fundamental Microbiology, University of Lausanne, 1015 Lausanne, Switzerland

**Keywords:** microbial communities, spatial organization, microbial interactions, synthetic communities

## Abstract

Microbial communities are frequently organized into complex spatial structures, shaped by intrinsic cellular traits, interactions between community members, initial growth condition or environmental factors. Understanding the mechanisms that drive these spatial patterns is essential for uncovering fundamental principles of microbial ecology and for developing applications. Using genetic engineering and synthetic microbial communities allows us to decipher how specific parameters influence spatial organization. In this review, we highlight recent studies that leverage synthetic microbial communities to deepen our understanding of microbial spatial ecology. We begin by exploring how initial conditions, such as cell density and relative species abundance, influence spatial organization. We then focus on studies that examine the role of individual microbial traits, such as cell shape and motility. Next, we discuss the impact of contact-dependent and long-range interactions, including metabolite exchange and toxin release. Furthermore, we highlight the influence of environmental factors on spatial dynamics. Finally, we address the current limitations of synthetic approaches and propose future directions to bridge the gap between engineered and natural systems.

## Graphical Abstract

**Graphical Abstract:**
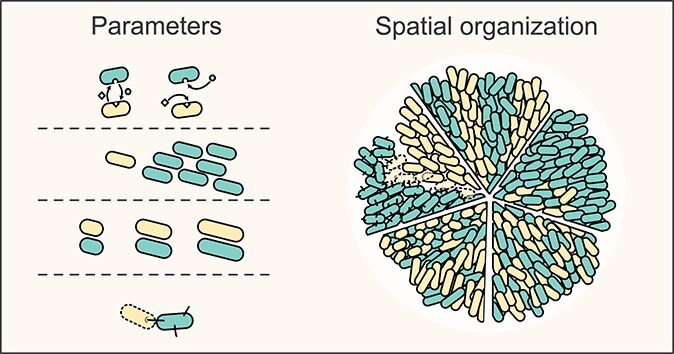

## Introduction

Microorganisms are ubiquitous on Earth, inhabiting virtually all ecological niches and playing pivotal roles in shaping ecosystems. They are integral to a wide range of processes with significant impact for agriculture, human health, and various technological applications [1–4]. Microbes rarely exist as monocultures, instead, they thrive within diverse communities, often forming intricate spatial structures. These spatial patterns can be partially attributed to the local chemical environment; uneven chemical landscapes form gradients and pockets with distinct properties cells respond by organizing themselves where their growth is favored [5, 6]. In turn, microorganisms actively reshape their environment by consuming and releasing molecules [7–9]. Beyond interacting with their environment, microbes also engage in intricate interactions with neighboring cells [10–16]. The dynamic interplay between biotic and abiotic interactions is responsible for the diverse spatial patterns observed in microbial communities.

Spatial organization in microbial communities often influences their functions and stability. For example, surface-attached communities exhibit greater long-term stability than liquid cultures, as spatial structure can separate interacting and non-interacting strains [17, 18]. Additionally, spatial organization enhances community resilience by physically excluding invaders and cheaters [19] and favors the coexistence of competitive species by spatially segregating them [20]. It also provides protection against external threats, such as phages [21] or disinfectants [22] by shielding sensitive species within the community. Similarly, spatial patterns modulate antibiotic resistance in a community of sensitive and resistant cells [23, 24].

One approach to studying microbial patterns is Fluorescence In Situ Hybridization (FISH) microscopy. This technique allows to detect and visualize the spatial organization of different microbial species within a natural community. FISH has been successfully applied to study microbial communities in complex environments such as on teeth, in the gut [12, 25, 26], or on plants [27, 28]. However, FISH is not well-suited for longitudinal studies, limiting its ability to capture dynamic changes in microbial communities over time. Moreover, by simply observing the final patterns it is difficult to disentangle the contributions of specific factors and interactions of each member of the community. In contrast, meta-omics approaches, such as amplicon sequencing, shotgun metagenomics, and metatranscriptomics, provide powerful tools to characterize the temporal dynamics of natural microbial communities. However, these methods still face significant challenges when it comes to retaining and analyzing the spatial organization of these communities [29].

A complementary bottom-up approach is to study pattern formation in simple and well-defined communities under controlled laboratory conditions [30, 31]. Such a deliberately assembled group of microbial species designed to answer specific questions or to perform distinct functions under controlled conditions is referred to as a synthetic microbial community. This approach allows for meticulous control over the environment, with parameters such as nutrient availability, temperature, or pH being carefully selected or even dynamically modified to suit the needs of a study. The initial conditions of the community, such as cell density or the ratio of different species, can also be controlled, further enhancing the ability to probe the underlying mechanisms driving community behavior. Moreover, the microbes can be genetically engineered, such as knocking out a gene, over-expressing it or introducing a heterologous one, to specifically test the effect of a property or interaction in isolation without confounding factors [32, 33]. Fluorescent labeling of the microbial strains adds another layer of control, enabling precise tracking of their position and abundance within the growing community [34].

To study pattern formation, synthetic communities are typically cultivated on solid surfaces, such as agar plates or patches, which provide a stable environment for tracking and analyzing patterns with a high degree of control. For example, in range expansion assays, a community, often fluorescently labeled, is inoculated on an agar plate and the spatial pattern formation during radial expansion can be observed [35]. Alternatively, microfluidics devices have been used to grow cells in chambers or traps where it is possible to track single cells over time, offering high resolution for recording spatial patterns and dynamics [36, 37].

In this review we highlight recent advances in understanding the spatial ecology of microbial communities, focusing on controlled, synthetic, and engineered communities that provide valuable insights into the fundamental principles governing microbial spatial patterns. We begin by reviewing studies that investigate how initial conditions, such as cell density and relative species abundance, shape emergent spatial patterns. We then examine how individual microbial traits, including cell shape and motility, influence spatial organization. Next, we highlight research exploring the role of contact-dependent cell–cell interactions in structuring microbial communities. We then explore long-range interactions, emphasizing diffusion-based exchanges like metabolite sharing. Following this, we discuss the impact of environmental factors. Lastly, we discuss the current limitations of these approaches and propose future directions to further advance the field and to bridge the gap between synthetic and natural systems.

## Initial conditions

**Figure 1:**
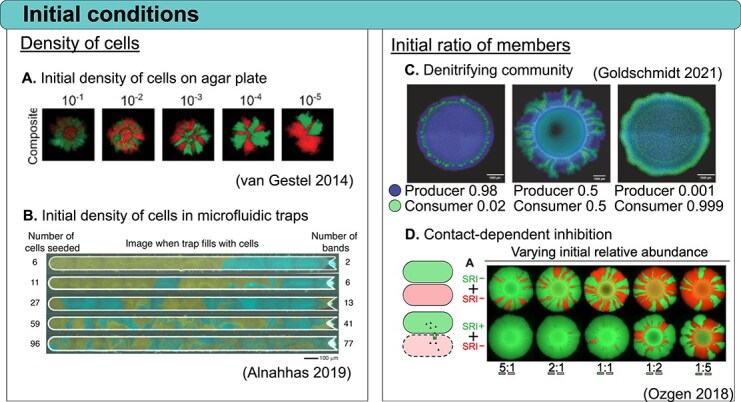
Effects of initial conditions on spatial patterns. (A) On agar plates, initial cell density influences spatial segregation, with higher starting densities leading to lower assortment levels between strains. Adapted with permission from [38]. (B) Similarly, in open trap microfluidics, initial cell density can influence spatial pattern formation, with higher starting densities leading to more intermixing. Adapted with permission from [39]. (C) Varying the initial ratio of cells in a producer and consumer community leads to distinct spatial patterns during expansion. Adapted with permission from [40]. (D) Short-range inhibition (SRI) causes strains to segregate more sharply at the expanding front, with higher initial ratios of one strain leading to a more pronounced separation between the two. Adapted with permission from [41].

Initial conditions are a key parameter that can influence the final spatial organization of a microbial community. (Fig. 1). The initial cell density, positioning, and frequency of each community member influence the resulting arrangement. For instance, in the range expansion of a co-culture of *Bacillus subtilis (B. subtilis)* strains initiated at low cell density, the two strains tend to segregate, while higher initial densities lead to more mixed patterns [38, 42] (Fig. 1A). Similarly, in *Pseudomonas aeruginosa (P. aeruginosa)* co-cultures, strains resistant to carbenicillin (an antibiotic, i.e. degraded extracellularly) outcompete susceptible strains at low densities due to a degree of spatial segregation. However, at higher initial densities, increased mixing allows susceptible strains to persist and even dominate the population [23]. Microcolonies inoculated at low density typically expand asymmetrically, with a single layer of cells, while denser ones are more symmetric with multiple layers of cells [43]. Similarly, in microfluidic chambers, the number of seeded cells directly correlates with the degree of mixing within the community [39] (Fig. 1B). Additionally, random positioning of founder cells on agar surfaces introduces variability in the final pattern and composition [40, 42].

The initial ratio of two strains in a community can also play a crucial role in determining the spatial patterns that emerge from range expansions. An example of this is a *Pseudomonas stutzeri (P. stutzeri)* cross-feeding community composed of a nitrite-producing strain and a consumer strain that relies on the produced nitrite for growth. When both strains start at an equal proportion (0.5), two distinct spatial arrangements emerge simultaneously within the same colony: “a producer first” and a “consumer first” pattern. However, when the producer strain is initially more abundant, only the “producer first” pattern appears. In contrast, when the consumer strain starts at a higher proportion, the “consumer first” pattern dominates (Fig. 1C) [40]. While this study highlights the impact of initial abundance on spatial organization, other systems show that initial ratios can also shape final strain frequencies. Müller and colleagues demonstrated that in a yeast community auxotrophic for two different amino acids, the initial ratio of the two members did not affect their final frequencies when they grew together in an obligate mutualistic relationship. However, when amino acids were added to eliminate the mutualism, the final frequencies of the cells directly depended on their initial proportions [44].

Finally, strain densities and frequencies can also influence the spatial pattern of communities employing antagonistic mechanisms, such as diffusible inhibitors or type VI secretion systems (T6SS). A higher initial density or skewed strain ratio often leads to one strain outcompeting the other, as spatial interference (such as toxin production) plays a more pronounced role. In contrast, at lower densities or more balanced ratios, the competition dynamics shifts, with the strains occupying different spatial regions where the strains can often co-exist [41] (Fig. 1D).

## Individual microbial properties

**Figure 2:**
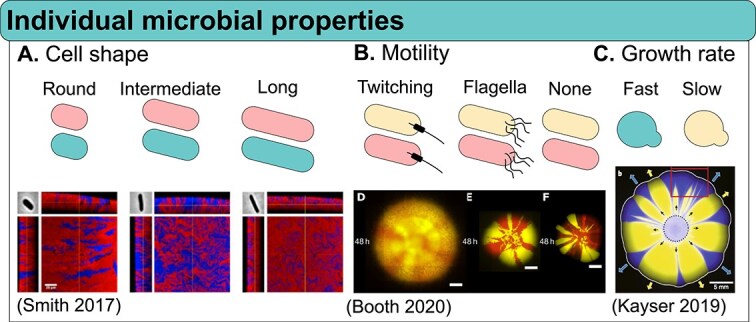
Individual microbial properties can influence spatial self-arrangement. (A) Cell shape influences spatial patterning in *E. coli* colonies, with strains of different aspect ratios showing variations in boundary smoothness and mixing complexity, ranging from fractal-like intermixing between the two strains with rod-shape to smoother group boundaries in more round strains. Confocal images of edge sections of each colony are shown alongside orthogonal projections. Adapted with permission from [45]. (B) Motility differences among cells influence spatial patterning in dual-species colonies, as shown by varying degrees of strain overlap and area colonization. Adapted with permission from [46]. (C) The spatial pattern of a yeast colony is influenced by growth rate differences, with slower-growing cells forming distinct monoclonal sectors that expand at varying rates and persist at the colony front before they are eventually expelled. Adapted with permission from [47].

Organisms have intrinsic properties that influence their spatial arrangements (Fig. 2). One of them is cell shape. For instance, two competing rod-shaped *Escherichia coli* (*E. coli*) strains form fractal patterns when grown together. However, when cells are engineered to be round, they produce smoother boundaries, and the mixing is increased between stains. Cells with an intermediate aspect ratio generate patterns with an intermediate level of mixing [45, 48] (Fig. 2A). When rod-shaped and round strains are mixed, distinct layered structures form within the biofilm: round cells tend to occupy the upper layers, while rod-shaped cells dominate the lower layers and edges. Given that nutrients are primarily accessible from one direction (e.g. from the agar surface), this spatial organization suggests that cell shape influences nutrient access. Consequently, cells with a specific shape may gain preferential access to resources, resulting in a fitness advantage for those better positioned. Elongated, filamentous cells have been shown to drive chiral pattern formation in *E. coli* colonies. When two isogenic strains grow on agar, they develop chiral or pinwheel-like patterns, with sector boundaries bending in a consistent direction. This macroscopic pattern arises from nonradial cell movement along the colony edge. The extent of colony chirality correlates with the presence of elongated, filamentous cells at the colony’s edge, revealing a connection between cell division and macroscopic colony patterning [49]. By shaping the spatial structure of cellular populations, chirality has been proposed as an ecological trait that influences competition and invasion dynamics [50].

Cell motility is another trait that influences pattern formation. Motile cells can colonize more space and interact with a higher number of neighboring cells. Booth and Rice showed that different motility genotypes lead to changes in mixing and area colonized (Fig. 2B). A mutant of *P. aeruginosa*, without a functional type IV pili—responsible for motility on surfaces—colonized a smaller area than the wild-type strain and exhibited significantly reduced mixing when co-cultured with wild-type *P. aeruginosa* [46]. Additionally, fast-swimming *Thiovulum majus* bacteria induce turbulence that attracts neighboring cells, leading to large-scale crystal-like packing on smooth surfaces [51]. Motility also plays a role in bacterial warfare, as it enables attackers to invade patches of target cells and to access fresh targets behind dead cells. For example, *P. aeruginosa* using a T6SS to kill a sensitive strain, was up to 10 000-fold more efficient than its nonmotile counterpart. This in turn influences the composition and spatial arrangement of the community [52].

Finally, growth rates also shape community arrangement. For instance, Kayser and colleagues studied the fate of fast- and slow-growing *Saccharomyces cerevisiae (S. cerevisiae)* lineages within a community (Fig. 2C). The two groups remained spatially separated. Although faster growing cells had a growth advantage, slower growing cells persisted at the expanding front, pushed forward by the fast-growers. This collective movement enabled slow-growers to produce more offspring than expected, boosting their chances of evolutionary survival [47]. Beyond the speed of growth, the lag time of founder cells influences the final pattern of a community, as cells that start growing earlier have an advantage in situations where cells are competing for space. This is because cells with shorter lag times begin expanding their microcolonies sooner, allowing them to occupy territory before neighboring cells can establish themselves, thereby reducing the available space for competitors and increasing their chances of outcompeting others [43].

## Contact-dependent interactions

**Figure 3:**
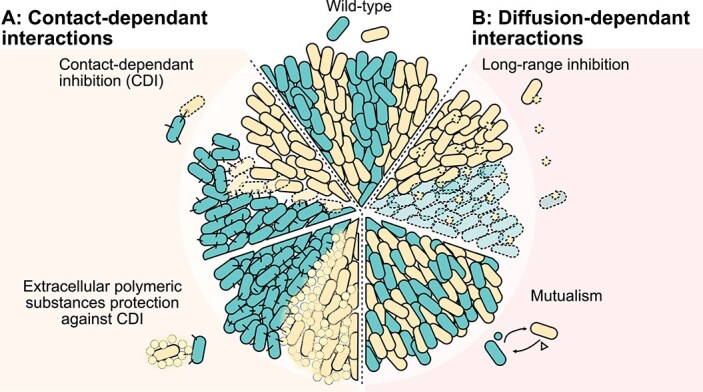
Interactions between cells affects spatial patterns. (A) Contact-based interactions. Contact-dependent inhibition shapes spatial patterns by promoting strain segregation and altering the relative abundance of strains at the expanding front [41]. EPS production, can create spatial structures by protecting both EPS-producing and non-producing strains from T6SS attacks, influencing survival and strain distribution in mixed colonies [53]. (B) Diffusion-based interactions. Long-range inhibition extends the spatial scale of interference, accelerating the exclusion of sensitive species compared to CDI [41]. Obligate mutualism alters spatial patterns by promoting the formation of smaller, intertwined patches in colonies [44, 54, 55].

Cells in microbial communities can have contact-mediated interactions, e.g. by inhibiting the growth of others through contact-dependent inhibition (CDI). This was studied in a synthetic community of *E. coli* strains, where one strain injected toxins into a sensitive strain. Ozgen and colleagues found that this interaction typically leads to a diminishing front of the sensitive strain, while the inhibitory strain overtakes the available space [41] (Fig. 3A). Similarly, T6SS can limit cell mixing in co-cultures. In a co-culture of *P. aeruginosa* strains—one with an active T6SS and the other sensitive to its attack—the sensitive cells are confined to isolated regions, limiting their expansion. Notably, contact-dependent inhibition occurs effectively in both high- and low-density bacterial populations [52, 56]. However, extracellular polymeric substances (EPS)-producing cells can form a physical barrier around sensitive cells, enabling their survival through a protective spatial arrangement. As a result, the community assembles into distinct “islands” of sensitive cells encased by EPS-producing strains and surrounded by T6SS-active cells [53] (Fig. 3A).

Phage predation reshapes bacterial community organization by shifting the fastest growth zone from the colony edge to its densely packed interior, resulting in straighter strain boundaries. This slows strain segregation and extends cell–cell contact, enhancing plasmid transfer via conjugation, including the spread of antibiotic resistance genes [57].

Finally, microorganisms also adhere to each other in a mutually non-harmful manner. For example, a cross-feeding community of *P. stutzeri* strains were found to form a “bubble-burst” pattern with a low level of intermixing. Repeating the experiment with engineered strains lacking type IV pili, resulted in a much more intermixed community [58], suggesting that pilus-mediated cell aggregation is responsible for this pattern. On the other hand, a growing colony of *E. coli* expressing the adhesin protein ag43 was shown to increase mixing [59]. It is interesting to note that cell–cell adhesion can lead to spatial structures even in liquid cultures. Engineered cells that express adhesins form large aggregates that settle in liquid cultures. By controlling the expression of pairs of adhesions proteins these aggregates can be modulated to display specific patterns (e.g. sequential layering, phase separated or well-mixed) and morphologies (fibrous, spheroid or porous) [60–62]. Such programmable aggregates of microbial communities are interesting for applications in environmental technology, agriculture, human health, and biotechnology. The study of adhesion systems also provides insight into the evolution of cellular aggregation and multicellularity. For instance, in experimental evolution with *E. coli* strains relying on each other for growth, cells formed multicellular clusters [63] and repeatedly acquired the ability to attach to others [64].

### Diffusion-based interactions

Members of a microbial community can interact with each other, even without direct contact, namely through the diffusion of molecules such as nutrients, signaling compounds, or toxins (Fig. 3B). These interactions significantly influence the spatial organization of microbial communities [65]. Kong and colleagues engineered synthetic two-strain consortia of *Lactococcus lactis* to exhibit specific social interactions including neutralism, cooperation, and competition [66]. The interactions were implemented using the antimicrobial and quorum-sensing peptide Nisin along with the antimicrobial peptide lactococcin A (lcnA). On agar plates, cooperating strains co-localized, competing strains became spatially excluded, and neutral strains were randomly distributed.

Diffusible molecules can be used as weapons: some microorganisms use diffusible toxins to inhibit or kill other cells at a distance. Celik and colleagues engineered both contact-dependent and diffusion-based inhibition in synthetic two-strain *E. coli* consortia, demonstrating that in unidirectional interactions, the extinction time of toxin-sensitive species at expanding fronts is inversely related to the interaction range. In other words, long-range inhibition was shown to be more effective than short-range interactions. However, in bidirectional interactions (i.e. strains acting with contact-based inhibitions versus strains attacking with diffusion-based inhibition), the outcome depended on the initial strain ratios, with long-range inhibition requiring high frequencies [41] (Fig. 3B). This was also confirmed in *P. aeruginosa* communities: a strain with tailocins long-range weapons defeated strains employing CDI only at high frequencies and densities. Consequently, their impact was also position-dependent; cells at the edges of colonies were less affected by tailocins due to lower cell density compared to the center [56]. Metabolite toxicity has also been shown to increase the time it takes for a population to segregate in space, thus maintaining higher strain diversity for a longer time [67].

Diffusible metabolites can also mediate mutualism. In communities with mutualistic metabolite exchange, spatial expansion can be challenging. As cells multiply, they tend to be surrounded by their own kind, limiting access to exchanged metabolites. Consequently, mutualistic communities are typically more mixed compared to competitive ones, where segregation is more pronounced. This is evident in amino acid auxotroph communities, where cells are well mixed in minimal media, but spatially segregated when amino acids are supplied, ceasing the need for the mutualistic interaction [44, 54, 55] (Fig. 3B). Other examples of increased mixing in mutualistic communities include denitrifying consortia and toluene-degrading communities [68, 69]. In addition, in polymer-degrading communities, diffusible breakdown products play a critical role in shaping spatial patterns. The cells that consume these breakdown products released by degraders actively modify their external environment through the secretion or removal of metabolites and compounds. These modifications can either positively or negatively influence the growth of degrader cells, ultimately affecting community assembly and function [70].

The diffusion properties of secreted goods strongly influence spatial patterns and, consequently, the success of cooperative behaviors in *B. subtilis* populations. During colony expansion, cells secrete compounds that facilitate surface spreading, such as the lipopeptide surfactin, the hydrophobin BslA, and exopolysaccharides (EPS), each with distinct diffusion characteristics. Surfactin diffuses broadly, allowing nonproducers to benefit from and integrate with producers at the colony front, resulting in mixed or chimeric patterns. In contrast, the limited diffusion of BslA and EPS confines nonproducers to the colony center, leading to spatial segregation and producer-dominated edges [71]. This diffusion-driven spatial organization similarly shapes cooperative strategies. For instance, genetic division of labor, where genotypes share EPS and protein fibers (TasA), enhances population mixing. However, when spatial assortment increases, such as through loss of motility, public good sharing diminishes, and overall biofilm productivity declines [72].

Despite the fact that small molecules can diffuse widely through space, if molecules are irreversibly taken up by cells in a dense microbial community, cells typically only interact with their immediate neighborhood through diffusion [73, 74]. This phenomenon was studied in synthetic communities of auxotrophic *E. coli* strains that exchange amino acids [55, 73, 75]. In these communities, the maximum distance at which an amino acid consumer can grow from a producer negatively correlates with amino acid uptake rates. Cells with highly efficient uptake absorb most of the available molecules before they can diffuse further, limiting the growth of consumers to close proximity. In contrast, when uptake efficiency is lower, amino acids diffuse farther, allowing consumers to grow at greater distances from the producers. Consequently, engineered strains with increased uptake rates resulted in greater community mixing during range expansions, as increased metabolite uptake reduced the distance between partner strains [55]. Similarly, quorum sensing molecules that are irreversibly taken up by cells can diffuse over long distances in sparse environments where they encounter few cells. However, in a densely packed environment, these molecules are primarily received by nearby cells [74].

Certain diffusing molecules can influence spatial patterns within microbial communities in multiple ways. For example, nitric oxide (NO) can either facilitate growth (as an energy source under anoxic conditions) or inhibit growth (through toxicity under oxic conditions) and promotes mutualistic or antagonistic behaviors depending on the micro-environment. Thus, in a gradient of oxygen, NO cross-feeding between producer and consumer strains produce complex spatial patterns driven by the contrasting roles of NO [76].

### Environmental conditions

**Figure 4:**
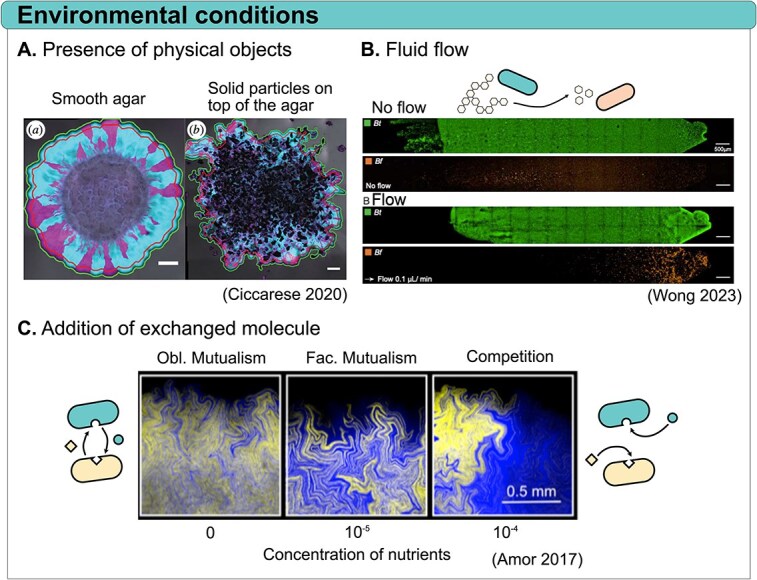
Effects of the environment on spatial patterns. (A) The presence of physical objects on a surface creates local deformities along the expansion frontier, which influence spatial pattern formation by altering the densities and orientations of interspecific boundaries (adapted with permission from [77]). (B) Fluid flow in the environment influences spatial arrangement by transporting metabolic by-products between species, impacting biofilm formation and altering the distribution of populations within the community. Adapted with permission from [78]. (C) The concentration of exchanged molecules (i.e. amino acids) alters spatial organization during range expansions by modulating the interactions between mutualistic strains, with higher concentrations promoting competition and larger single-strain patches, while lower concentrations support stable mutualism and smaller patches. Adapted with permission from [54].

Microbes interact not only with one another but also with their environment, and in some cases, these interactions significantly influence the spatial self-organization of microbial communities. While range expansions are typically studied on smooth surfaces, the presence of physical objects on top of the agar was shown to have a substantial impact on pattern formation. Small objects were shown to disrupt the circular colony expansion, as well as alter boundaries between strains and increase mixing [77] (Fig. 4A). Similarly, the presence of fungal hyphae in a bacterial co-culture enhances spatial intermixing and dispersion in both competitive and cross-feeding populations. This occurs as bacterial cells disperse along the hyphal network, counteracting the population segregation typically observed at the expanding frontiers [79]. Dispersal is also influenced by the properties of the surface on which cells grow. For instance, cells tend to disperse more effectively on softer surfaces, such as those with a low agar concentration, which enhances their motility and swimming ability. In a community composed of *P. aeruginosa* strains, this change of environmental property was shown to significantly increase the final expansion size without affecting intermixing [46].

The size of the available space in which cells grow can also influence the spatial arrangement and composition of the community. For example, Alnahhas and colleagues grew a community of engineered *E. coli* strains in microfluidic devices with cell-trapping regions (traps) of different sizes. They found that in small traps, the relative proportion of each strain fluctuated wildly, while in large traps, the relative ratio of strains was stable [39].

Another factor playing a role in the self-organization of bacterial communities is the temperature, where non-optimal temperatures reduce growth rates, resulting in smaller colony expansion. In addition, temperature changes in *E. coli* lead to longer, more filamentous cell shapes, which, as discussed earlier, promote the formation of more chiral colonies [49].

In environments with flow, such as the gut or rivers, the distribution of molecules not only depends on diffusion but also on the transport by flow, which is generally in a unilateral direction. Thus, fluid flow impacts the composition and spatial organization of communities by shaping gradients of nutrients. For example, two members of the human gut microbiota, *Bacteroides thetaiotaomicron* and *Bacteroides fragilis*, were grown in a microfluidic channel. While *B. thetaiotaomicron* can metabolize dextran, *B. fragilis* cannot; however, it can grow using the metabolites released by *B. thetaiotaomicron*. In the absence of flow, both species co-colonized the entire surface of the channel. In contrast, under unidirectional flow, the *B. fragilis* population shifted downstream due to the transport of metabolites produced by *B. thetaiotaomicron* with the flow (Fig. 4B) [78].

Environmental conditions are often not constant but change dynamically [80]. Cellular interactions can change dramatically when environmental perturbations occur. This is evident with amino acid auxotrophs whose interactions change depending on the presence or absence of external amino acids [44]. In such cases, spatial organization becomes crucial for maintaining metabolic interactions within the community. For example, when amino acids are removed in a microfluidic setup, only cells in the immediate proximity of their metabolic partners can rapidly regrow [81]. Environmental changes can therefore reshape spatial patterns by influencing which cells regrow first, ultimately affecting the distribution and dominance of different strains within the community. Similarly, fluctuating pH in oxic environments has been shown to influence spatial pattern formation in a *Paracoccus denitrificans* community: The accumulation of nitrogen oxide intermediates, particularly nitrite and NO under low pH conditions, reduced growth, leading to increased spatial intermixing between subpopulations at the colony periphery. This increased intermixing preserved greater genetic diversity within the population, creating localized niches and broadening the evolutionary potential of the community [82].

In co-cultures of two metabolically interacting *P. stutzeri* strains, environmental fluctuations—alternating between anoxic conditions (which promote mutualistic interactions) and oxic conditions (which induce competition)—strongly influence spatial organization. These shifts give rise to rare, localized clusters where co-occurrences of both strains persist despite changing conditions. These patterns stabilize community composition by supporting localized niches, demonstrating how environmental changes can drive the emergence of spatial structures that ensure the persistence and functionality of cross-feeding microbial communities [83].

Environmental conditions can also be leveraged to regulate interactions and pattern formation in synthetic and engineered communities. Notably, optogenetics enables the optical modulation of specific cells by introducing proteins with light-sensitive domains linked to biological functions [84]. Light-controllable cells allow for precise, dynamic, and spatial regulation, providing a powerful tool for shaping community organization. For example, engineered *E. coli* with optogenetic adhesin expression enabled high-resolution light-regulated biofilm formation [85]. Optogenetics has also been used to control the spatial organization in a synthetic yeast community by modulating interactions between its members. Le Bec and colleagues engineered yeast cells to act as cooperators under light exposure, converting sucrose into hexose (a public good which can be consumed by all the cells), but to behave as cheaters in the dark, consuming hexose produced by cooperators. By selectively illuminating specific regions, they spatially restricted the behavioral transition. Light-induced shifts in behavior drove the emergence of distinct spatial patterns within the community and allowed to decipher the length scales that set the domain size of both cooperators and cheaters [86].

### Limitations and future directions

Spatial self-organization plays a crucial role in the functioning, ecology, and evolution of microbial communities, shaping their functions, stability, and adaptive potential. Studying these spatial dynamics provides insights into microbial behavior in engineered and natural communities.

Pattern formation is influenced by a complex interplay of factors. Understanding these processes has been greatly facilitated by the use of engineered strains and synthetic communities with defined properties and behaviors. By precisely tuning environmental conditions or initial community setups, these simple systems have become powerful tools for unraveling the contributions of individual factors and to decipher the fundamental principles that shape microbial organization [87–89].

While the simplicity of engineered and synthetic communities in controlled environments is a key strength of this approach, it also presents a significant challenge when translating findings to natural systems. Synthetic communities generally lack the complexity of real-world microbial communities, particularly in terms of species diversity, genetic variability and the intricate web of interactions [90]. As a result, these engineered model systems cannot fully capture the dynamic and multifaceted nature of natural microbial ecosystems. Notably, most studies reviewed here rely on a limited set of model organisms, such as *E. coli, S. cerevisiae*, and *Pseudomonas* species, with communities typically composed of only two strains or species. Moreover, studies typically focus on isolating and examining a single aspect of microbial interactions at a time, which can oversimplify the complexity of natural ecosystems. The effects of different parameters or interactions may not be additive but rather interact in nonlinear and often unpredictable ways [91, 92]. The interplay between multiple factors can lead to emergent behaviors that are not captured when examining individual variables in isolation. Another critical limitation of synthetic systems is that they rarely study host-associated microbial communities, therefore not taking in account the interaction between microorganisms and their host. In many natural environments, microbial spatial arrangements are deeply influenced by host-related factors, which can modify the behavior and organization of microbial populations [25, 76, 93–95].

To bridge this gap between synthetic and natural communities, future research should therefore incorporate more complex systems and combine multiple parameters simultaneously to better mimic natural environments [96]. One possible approach would be to increase the relevance and complexity of synthetic microbial communities. In recent years, several synthetic consortia have been deliberately constructed to reflect key taxa, gene combinations, keystone species, or interaction motifs frequently identified in amplicon and metagenomic surveys [97]. These communities represent a range of ecosystems, including the mouse gut microbiome [98, 99], the insect gut [100–102], the plant phyllosphere [103], and soil environments [104]. Studying spatial organization within these synthetic systems under controlled conditions could help bridge the gap between very simple synthetic communities composed of strains of one model organisms and the complexity of natural microbiomes.

Another promising approach to achieve this is the increased integration of microfluidics technology [105]. Notably, several high-throughput platforms have been developed to study (higher-order) interactions within microbial communities [106–109]. While most high-throughput devices currently focus solely on growth analysis, some have the potential to examine spatial organization, albeit typically at scales below 100 *μ*m. Microfluidic systems also offer precise control over environmental conditions while enabling real-time observation of microbial behavior. For instance, the MISTiC (Mapping Interactions across Space and Time in Communities) platform was designed to investigate how defined spatial structures and temporally varying environmental signals influence microbial community properties [110]. The “microfluidic palette” can simultaneously generate multiple spatial chemical gradients within a single chamber [111]. Another platform, which utilizes aerogel matrices, systematically varies combinations of environmental factors, including temperature, CO_2_, light, and nutrients, to assess their impact on the system under study [112]. Furthermore, the development of microfluidic organ-on-a-chip-devices closely mimic the native tissue topologies and physiological aspects [113]. These advanced models can provide a physiologically relevant platform for studying host–microbe interactions in controlled environments. Together, these approaches allow to approximate the environmental complexity of natural ecosystems. Incorporating such strategies with the study of synthetic communities could significantly enhance our ability to investigate how microbial communities respond to and organize within dynamic, multidimensional environments.

To improve further the ecological relevance of synthetic insights, a stepwise validation framework could help bridge the gap between controlled systems and natural ecosystems. One conceptual path forward might involve embedding well-characterized synthetic consortia into microcosms, such as soil [104] or aquatic environments [114], enabling to track how synthetic consortia persist, adapt, or fail within diverse and competitive microbial networks. Of course, when focusing on spatial organization, a key technical challenge lies in accurately resolving it within the complex context of a microcosm.

An additional promising strategy is the use of biosensors. These are genetically-engineered microbes that detect and report on specific environmental conditions [115, 116]. For example, they can be tailored to respond to quorum-sensing molecules or metabolite production, enabling the monitoring of the spatial distribution of these signals within complex environments such as the gut [117–120].

Another interesting approach to bridge the gap between correlative, descriptive studies of microbiome composition—often limited in taxonomic and mechanistic resolution—and molecular studies focused on causality in simplified synthetic systems is the N + 1/N-1 concept. This framework aims for the systematic addition or removal of individual community members within their native habitat, enabling researchers to probe both the outcomes and underlying functional processes of microbial interactions [121].

In addition, computational models are an essential tool for understanding and predicting microbial community behavior across diverse scenarios. While in this review we focused on the experimental results, many of the cited studies incorporate mathematical models and simulations to interpret their findings. Beyond merely reproducing experimental outcomes, models enable the simulation of interactions that are challenging to observe experimentally and provide a powerful platform to efficiently screen a wide range of parameters. Refining these models by incorporating complex variables—such as mechanistic descriptions of molecular and cellular processes—and integrating cell-environment interactions can further enhance their capacity to simulate and predict emergent system properties and community dynamics [122–124].

Insights from studies on the spatial organization of microbial communities not only illuminate fundamental aspects of community formation and function, but also have practical implications. As highlighted by several studies discussed in this review, spatial structure can significantly influence pathogenicity as well as susceptibility to antibiotics and phages [21, 57]. Incorporating spatial considerations into the design of antimicrobial therapies could therefore enhance treatment efficacy and improve strategies to combat resistance. By targeting or disrupting the physical architecture of microbial communities, therapeutic interventions may become more effective [125]. In other contexts, engineering specific spatial arrangements can be used to promote beneficial microbial functions. For instance, in bioremediation, a core–shell spatial configuration enabled a community to simultaneously degrade pentachlorophenol (PCP) and remove mercury—functions that neither species could perform alone or when randomly mixed. In this system, *Sphingobium chlorophenolicum*, which degrades PCP but is sensitive to mercury, was positioned in the core, surrounded by a shell of protective mercury-reducing *Ralstonia metallidurans* [126]. In bioproduction, distributing metabolic functions across different strains or species has been shown to enhance yield. Ensuring the stable coexistence of these community members can be achieved through engineered interactions or, alternatively, by controlling their spatial organization [127, 128]. Spatial structuring may also help optimize agriculture and food production processes, in which spatially organized microbial communities play a crucial role, e.g. in cheese making [129] or in soil inoculation with beneficial microbes [130]. Finally, in the emerging field of engineered living materials, precise control over the spatial arrangement of functionally distinct strains or species is an important design goal [131].

In conclusion, studying the spatial organization of microbial communities is crucial for understanding, predicting, and ultimately engineering their interactions, stability, and functions. As outlined in this review, studying synthetic and engineered consortia provides a powerful approach for dissecting how specific parameters shape spatial structure. However, further research is needed to translate these insights from simplified synthetic systems to the complex realities of natural microbial communities.

## Data Availability

Data sharing not applicable to this article as no datasets were generated or analyzed during the current study.
